# Deep-Learning Solution Providing Molecular Marker Subtyping of Breast Cancer Whole Slide Images: Protocol for a UK Clinical Service Evaluation Study

**DOI:** 10.2196/76785

**Published:** 2026-06-16

**Authors:** Elizabeth Walsh, Dildar Rathore, André Geraldes, Salim Arslan, Andrew M Hanby, Rebecca Millican-Slater, Michael Drummond, Olorunda Rotimi, Narender Kumar, Pahini Pandya, Nicolas M Orsi

**Affiliations:** 1 Leeds Institute of Medical Research Faculty of Medicine University of Leeds Leeds United Kingdom; 2 Histopathology Department St James's University Hospital Leeds United Kingdom; 3 Panakeia Technologies Ltd Cambridge United Kingdom; 4 Centre for Health Economics University of York York United Kingdom

**Keywords:** digital pathology, deep learning, artificial intelligence, AI, breast cancer, service evaluation, protocol

## Abstract

**Background:**

As the histopathology workforce continues to struggle and service demand continues to increase, it has become prudent to consider viable avenues to try to alleviate diagnostic workload burden. One such avenue is computer-based technologies (CBTs). Breast cancer (BC) is the most common malignant neoplasm in the United Kingdom and requires additional testing for estrogen receptor (ER), progesterone receptor (PR), and human epidermal growth factor receptor-2 (HER2) status at the time of histological diagnosis. This makes BC diagnostics a promising candidate for the application of an efficient CBT. However, for clinical acceptance, these technologies must prove that they work within a real-life diagnostic environment.

**Objective:**

We present a study protocol for a prospective clinical service evaluation aimed to validate a UK Conformity Assessed–marked CBT’s ability to provide ER, PR, and HER2 results for invasive BCs from scanned hematoxylin and eosin-stained whole slide images.

**Methods:**

This protocol has been designed to use and mimic a preexisting digital pathology workflow within a National Health Service tertiary referral cancer center without disrupting normal patient care. Eligible cases are identified prospectively through the laboratory information management system, and their whole slide images are extracted from the clinical digital workflow. After verification of national data opt-out status and the exclusion of appropriate cases (N=400 analyzable cases), these cases are analyzed on a dedicated computer in parallel to the existing clinical workflow by a UK Conformity Assessed–marked deep learning–based CBT in a separate environment, providing results for ER, PR, and HER2 status. These results are compared to the ER, PR, and HER2 status reported on the corresponding pathology report. To evaluate the CBT’s performance, a range of accepted concordance measures will be applied, including specificity, sensitivity, false-positive rate, false-negative rate, positive predictive value, and negative predictive value. Moreover, time stamps representing the duration of image analysis will also be collected.

**Results:**

This study started in April 2025. There are no results to present, as this paper focuses on study design, and results have yet to be generated. As of March 2026, overall, 366 potentially analyzable cases have been collected. The anticipated end date of the study is May 2026 (400-case target). Results will be presented in a separate publication.

**Conclusions:**

This design assesses a CBT within a clinical environment while effectively eliminating any unwanted effects on patient care. This type of service evaluation provides a useful step to establish confidence in a CBT before trialing its effect on patient care. It also offers the opportunity to support interventional randomized controlled trials, health economic evaluations, and usability studies. This protocol will hopefully prove useful to others who wish to conduct a similar service evaluation at their own institution.

**International Registered Report Identifier (IRRID):**

DERR1-10.2196/76785

## Introduction

It is now well recognized that the histopathology workforce, both in the United Kingdom and globally, is in crisis [1]. The UK Royal College of Pathologists’ 2018 workforce census revealed that only 3% of departments were sufficiently staffed to meet clinical need [2]. The report also highlighted that a quarter of histopathologists were older than 55 years, and the workforce in England would fall to 74% of its current level if all these pathologists were to retire within 5 years. Additionally, the census estimated that there is a UK-wide £27 million (CAD $50.09 million=US $36.68 million) annual spend compensating for staffing shortfalls through locums and outsourcing work. The census also acknowledged the increasing use of additional molecular and genomic testing in diagnostic work. This predicament is exacerbated by the increasing cancer burden. In this respect, Cancer Research UK reported that more than 385,000 new cancers were diagnosed annually in the United Kingdom between 2017 and 2019 [3]. This is predicted to increase to approximately 506,000 by 2038 to 2040 [4]. This increase would place unparalleled pressure on an already stretched histopathology service, potentially affecting patient care.

Digital pathology is an area that the Royal College of Pathologists has acknowledged could help alleviate this problem by enabling more efficient working [2]. In fact, pathology departments throughout the United Kingdom are starting to digitize, creating an opportunity to explore the merit of adjunct computer-based technologies (CBTs). These could lighten the workload of both pathologists and pathology departments by replacing or assisting with time-consuming tasks. For example, the first and currently only Food and Drug Administration–approved artificial intelligence (AI)–based pathology software Paige Prostate [5] can aid pathologists by highlighting areas suspicious for carcinoma in prostate biopsies [6-8], apparently reducing a pathologist’s review time of a full set of cases by 65.5% [6]. In fact, a service evaluation, the Artificial Intelligence for Cellular Pathology Transformation in Prostate Practice project based in Oxford, United Kingdom, has been conducted to assess the use of Paige Prostate, helping histopathologists with prostate biopsy evaluation [9,10].

Another common malignant tumor where time savings could be invaluable is breast cancer (BC). In the United Kingdom, data from 2017 to 2019 showed that BC was the most common malignancy seen in the country over that period [11]. Additionally, all newly diagnosed invasive BCs in the United Kingdom undergo testing for estrogen receptor (ER), progesterone receptor (PR), and human epidermal growth factor receptor-2 (HER2) status to determine molecular subtype and guide treatment [12]. ER and PR testing involves immunohistochemistry [12], while HER2 testing requires immunohistochemistry with or without in situ hybridization (ISH) [13]. These ancillary tests generate more work and cost for histopathology laboratories in terms of specimen preparation and staining as well as commandeering pathologists’ time to score and report the results. In this setting, any technology that could alleviate this time burden would be welcome. This would be particularly beneficial if it could extract such diagnostic information from hematoxylin and eosin (H&E)–stained whole slide images (WSIs) alone, as H&E-stained tissue interpretation is already the first, essential step in a pathologist’s diagnostic assessment. Akin to Paige Prostate, the technology company Ibex has been awarded UK-based funding to trial its BC diagnostic platform in a number of UK hospitals [14], although this technology focuses on BC detection on H&E-stained WSIs rather than molecular subtype profiling [15].

While CBTs have become an attractive area for research scientists and manufacturers, no such technology has translated into the active clinical space in the United Kingdom. The underlying reasons are multifaceted, including the need for significant validation [16,17], cost implications [16-18], and the lack of defined minimal performance levels acceptable to pathologists [17]. It has been proposed that, ideally, robust validation for CBTs requires a multicenter clinical trial with a cost-benefit analysis [16]. In this regard, the United Kingdom’s National Cancer Research Institute Cellular Molecular Pathology Initiative and the British In Vitro Diagnostics Association published a road map for bringing AI into routine clinical pathology practice [19]. This recommends that a trial akin to phase I (analytic validation) and phase 2/3 (clinical validation) in drug development be carried out to facilitate the regulatory approval and clinical use of these technologies. In addition, they describe phase I studies as including a health economic assessment. Furthermore, they provide a study comparing a tool for grading Ki-67 in neuroendocrine tumors entering a department during a set time frame with pathologists as an example of a “clinical (phase 2/3) validation”.

To help bridge the gap between development and clinical adoption, we have designed a protocol for a prospective clinical service evaluation of a UK Conformity Assessed (UKCA)–marked CBT implemented within digital pathology. This study evaluates a CBT’s ability to provide the molecular subtype (ER, PR, and HER2 status) of invasive BCs on biopsy specimens within an operational, digitized histopathology department. This evaluation has been purposefully designed to run alongside routine diagnostic practice and not interfere with patient management. This ensures that the CBT can be assessed in a clinical environment without direct impact on patients. We believe that this is an essential step to build confidence in these technologies before they are used to inform patient care. The CBT to be tested in our specific study is the UKCA-marked, deep-learning technology PANProfiler Breast (Panakeia Technologies Limited).

The design of this service evaluation is shared herein to provide other investigators with a reference point to conduct other similar studies to help bring CBTs into the digital pathology space. The aim of this service evaluation is to validate the technology’s performance in the molecular subtyping of invasive BCs from H&E WSIs of BC biopsy specimens in a real-life clinical environment.

## Methods

### Service Evaluation Design and Setting

**Figure 1:**
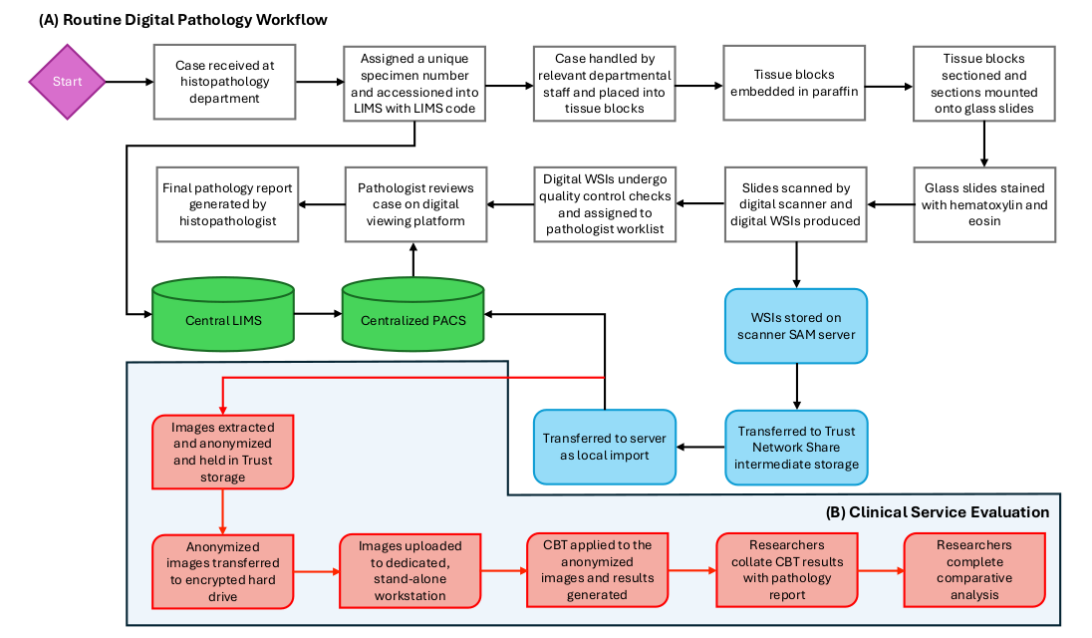
Demonstration of the clinical service evaluation design and its integration into a digital pathology workflow. (A) Steps in the routine digital pathology workflow. General specimen processing and reporting are shown in white. Handling of digital images is shown in green. Centralized digital systems are shown in blue. (B) Steps in the clinical service evaluation. CBT: computer-based technology; LIMS: laboratory information management system; PACS: picture archiving and communication system; SAM: server application monitor; WSI: whole slide image.

This service evaluation has been designed to be carried out within a pathology department with a pre-established digitized workflow. Our study will take place at St James’s University Hospital, Leeds Teaching Hospitals National Health Service (NHS) Trust (LTHT), United Kingdom. We have attempted to simulate how a CBT could integrate into the journey of a breast biopsy specimen suspicious for malignancy through this specific digital workflow. As mentioned, this evaluation is noninterventionist and runs parallel to the existing clinical pathway. It avoids interrupting or affecting laboratory processes, clinical reporting, or patient management. The generic steps of this clinical service evaluation are presented in Figure 1. To ensure minimal disruption to clinical services, all trial work will be conducted by a dedicated research team.

### Identification of Cases

**Figure 2:**
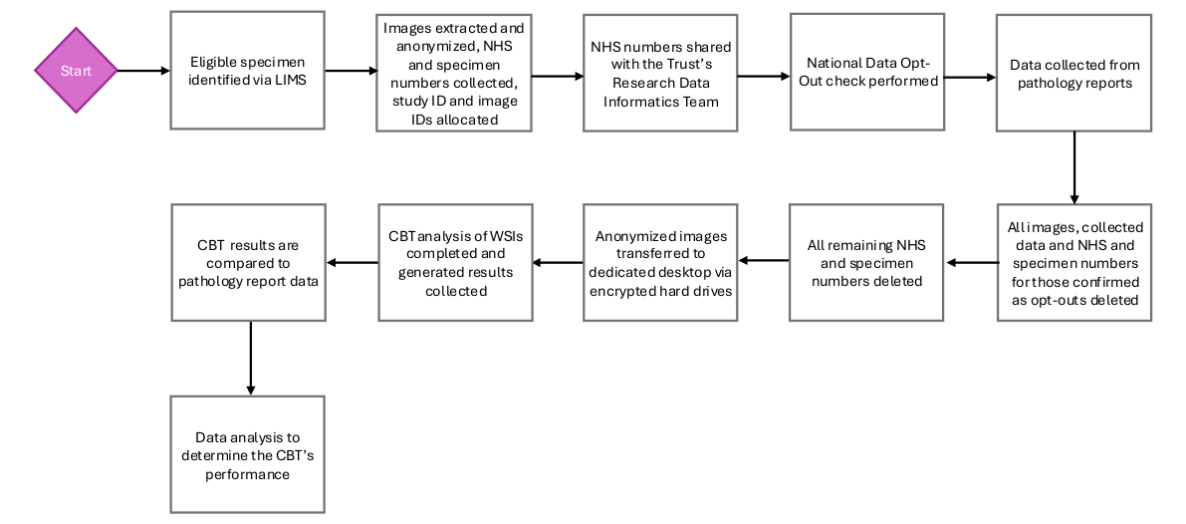
Data handling through the clinical service evaluation. CBT: computer-based technology; LIMS: laboratory information management system; NHS: National Health Service; WSI: whole slide image.

Eligible cases (N=400) will be identified prospectively as they are logged in at the histopathology department. At LTHT, cases are routinely assigned a unique Trust-specific specimen number and categorized using the local laboratory information management system (LIMS) codes. Breast biopsies that are suspicious for malignancy at the time of sampling are assigned one of a set of specific LIMS codes. Researchers will search the LIMS using this set of specific codes to identify eligible cases (Figure 2). Generic inclusion criteria include all patients with a breast biopsy specimen processed at St James’s University Hospital. Exclusion criteria are samples in line with the device’s indications for use. These include (but are not limited to) noncarcinoma malignancies (eg, angiosarcomas), malignancies not arising from breast tissue (eg, cutaneous carcinomas arising from breast skin), and those confirmed not to have invasive malignancy. These exclusions will be performed at the time of data collection from pathology reports. Applying these criteria, convenience sampling will be used to (1) simulate the histopathology workflow as closely as possible and (2) ensure that all relevant or eligible cases are captured during the study period. When identified, the case’s specimen number and NHS number will be collected, and a unique, anonymized study number will be allocated. The digital WSIs for a case will also be extracted (refer to the Extraction of Digital Images section), and an anonymized image number will be assigned that is aligned with the corresponding study number.

### Extraction of Digital Images

Once all potential cases are identified for the service evaluation, their images will be extracted and saved securely on the Trust’s IT storage (Figures 1-2). This design uses WSIs scanned as part of a histopathology department’s pre-established workflow (Figure 2). Briefly, researchers will manually access and duplicate relevant WSIs, saving them on the Trust’s secure IT server. Images will be anonymized using an SVS-deidentifier tool (Pearcetm); licensed by the Massachusetts Institute of Technology), which removes patient metadata without affecting the WSI. To remove the Trust-specific specimen number, researchers will manually rename the image files with the unique, anonymized image number assigned to the case.

### NHS National Data Opt-Out Checks

After the identification of eligible cases and corresponding image extraction, National Data Opt-Out Scheme (NDOS) checks will be completed (Figure 2). The NHS England NDOS offers patients the option to opt out of their data being used for research [20]. At LTHT, the Research Data and Informatics Team (R-DIT) will perform NDOS checks to ensure patients who have opted out are identified and have their wishes honored. To perform these checks, R-DIT requires patient NHS numbers. The research team will securely transfer the NHS numbers of the samples identified for the service evaluation to the R-DIT. R-DIT will then inform the research team which cases can or cannot be included in the study. All cases that have opted out will have all data and images collected up to that point deleted. Additionally, the NHS numbers for all cases will be deleted as they will no longer be required.

### Data Collection From Pathology Reports

To determine the accuracy of the CBT, researchers will collect ER, PR, and HER2 data from authorized pathology reports (Figure 2). The cases that are subsequently excluded because of NDOS compliance will have their data deleted before any analysis. At LTHT, breast specimens are reported by breast subspecialist histopathologists, and the results reported for ER, PR, and HER2 status in these reports will be used as the ground truth. This data collection will take place before the CBT results are generated by the research team and will not be shared with the technology providers. This keeps the evaluation fully blind. For completeness, the type of specimen will also be collected, for example, core biopsy or vacuum-assisted biopsy. Once this information has been collected from the pathology reports, the specimen numbers for all cases will be deleted. This removes any link back to the patient, which is particularly important when starting to analyze the CBT’s performance.

### Transfer of Anonymized Digital Images to a Dedicated Workstation

A dedicated desktop PC will be established to run the CBT. The CBT will be installed before eligible samples are identified. Once set up, the anonymized WSIs will be uploaded to this dedicated desktop via 256-bit encrypted keypad external hard drives and stored securely (Figures 1-2). This desktop will be located within the NHS Trust intranet; however, it will not be connected to the LIMS or the electronic patient record system. This will help ensure there is no crossover between the service evaluation and standard patient care.

### The CBT To Be Evaluated

The CBT to be used in this service evaluation is PANProfiler Breast, a UKCA-marked, deep learning-based technology that leverages computational methods to analyze H&E-stained BC WSIs. Specifically, it extracts histomorphological features associated with ER and PR expressions as well as HER2 status. This has been described in detail elsewhere [21].

### The CBT’s Analysis of Anonymized Digital Images

Once the anonymized WSIs have been uploaded to the dedicated PC, the research team will use the CBT and analyze the images (Figures 1-2). This will generate results for ER, PR, and HER2 status, and these will be recorded by the researchers. Time stamps pertaining to the time and date of file upload and the generation of results following image analysis will also be collected. ER, PR, and HER2 results will be compared to the ground truth. This image analysis will only be conducted after the data from the corresponding pathology report have been collected and the specimen number deleted. Because we do not intend to influence patient care, we prevented the CBT results from linking back to the patient in this way. After the image analysis has been completed for a case, the images will be deleted from all hard drives. This approach ensures adherence to Trust Information Governance requirements and prevents the images from being mistakenly analyzed more than once.

### Data Analysis

To validate the CBT’s diagnostic accuracy in this clinical service evaluation, concordance will be established with the ground truth (ie, the pathology report). Measures including sensitivity; specificity; false-positive rates and false-negative rates; and positive and negative predictive values for ER, PR, and HER2 will be calculated based on the formulae presented in Table 1. The time difference between the 2 time stamps collected will also be calculated. From this, a range and median time for the CBT’s result generation will be quantified.

**Table 1:** The standard diagnostic metrics used to measure the performance of PANProfiler Breast.

Performance metric	Definition
Concordance	Number of concordant cases/total number of cases with definitive PANProfiler result
Sensitivity	TP^a^/TP+FN^b^
Specificity^c,d^	TN^c^/TN_FP^d^
Positive predictive value	TP/TP+FP
Negative predictive value	TN/TN+FN
False-negative rate	FN/TP+FN
False-positive rate	FP/TN+FP
Test replacement rate	Number of cases with definitive PANProfiler result^e^/total number of cases
Complete test replacement rate	Number of cases with definitive PANProfiler results*for all marks/total number of cases

^a^TP: true positive.

^b^FN: false negative.

^c^TN: true negative.

^d^FP: false positive.

^e^Definitive PANProfiler result for HER2 is “negative,” and definitive PANProfiler result for estrogen receptor or progesterone receptor is “positive” or “negative.” The table is taken from the supplementary material of our previous publication [21].

### Ethical Considerations

On consultation with and review from the local Research and Innovation team, it was decided that because this study is a service evaluation, ethics approval is not required [22]. This was confirmed using the Human Research Authority Research Decision Tool [23]. Explicit patient consent was also deemed unnecessary, as researchers were considered to be members of the direct clinical care team. The lack of participants means that compensation disclosure does not apply. Additionally, this protocol prioritizes minimizing disruption to the clinical diagnostic pathway and interfering with patient care. Using a preexisting IT infrastructure and conducting our analysis outside of the direct specimen pathway, we help to limit any disturbance to the clinical service provided by the pathology department. Moreover, using a separate dedicated PC, we prevent the CBT’s interference with the reporting system and subsequently patient care. This is enhanced by the fact that connections between the WSIs to be analyzed and the patient are removed before the CBT is applied. This is especially important in the advent of discordant results between the CBT and the current standard of care.

With regard to patient confidentiality, only necessary patient identifiable data will be collected and then deleted at the earliest opportunity. In fact, NHS numbers are only collected to facilitate honoring patients’ choices with regard to the NDOS check. This process is necessary to allow patients’ opt-out wishes to be respected and upheld. The specimen numbers facilitate the collection of data from the corresponding pathology reports, and these are Trust-specific. From this point, all data collected will be anonymized. The WSIs are also anonymized at the time of extraction, with an additional quality control check put in place. Furthermore, access to the data and WSIs collected as part of this service evaluation will be restricted to the research team.

In this vein, another consideration throughout this design is data security. WSIs for the service evaluation will be kept on the NHS Trust secure servers in a dedicated folder and subfolders. Their transfer to the dedicated CBT workstation will be enabled by an encrypted hard drive, which will be stored securely on Trust premises. As mentioned previously, all images will be deleted from the hard drive after the CBT analysis is complete. The other data collected (including NHS numbers) will be stored on the Trust’s Microsoft OneDrive system, with access restricted to the research team. When transferring NHS numbers to R-DIT for NDOS checks, a secure NHS email will be used.

## Results

The study started in April 2025. There are no results to present to date, as this paper is focused on study design, and final results have yet to be generated. As of March 2026, 366 potentially analyzable cases have been collected, and the anticipated end date is May 2026. This study will be considered complete once the target 400 cases have been analyzed. Given the prospective nature of this study, the duration/achieving this target will depend on the variability of routine clinical throughput. The results of this evaluation will be the subject of a separate publication in which any deviation from this protocol will be described, as appropriate.

## Discussion

### The Protocol in a Wider Context

This protocol details a clinical service evaluation for validating the ability of a diagnostic CBT within a digital pathology workflow. To the best of our knowledge, no CBT has been approved by the National Institute for Health and Care Excellence (NICE) for use within digital pathology. In fact, a NICE medtech innovation briefing on Paige Prostate published in 2021 highlighted the absence of prospective, UK-based evidence [24]. General comments provided by experts read that “prospective use and audits would be important to inform how valuable the technology could be in practice,” and the document referenced the Oxford-based project as a “recent and ongoing” study. This protocol sets out such a study in the BC field. It is sufficiently flexible to be adapted to ever-evolving diagnostic parameters in clinical practice as well as ongoing updates to technologies. Clinical service evaluations after such updates are critical to ensuring that high levels of performance are maintained in the face of changes, for example, when the definitions of HER2 status were updated in 2023 in the United Kingdom to incorporate the emergence of HER2-low BCs [25].

Consideration should be given to what the next steps may be from a service evaluation such as this to full clinical adoption. Given the different digital infrastructures within NHS Trusts across the United Kingdom, service evaluations should be conducted for any given technology within each specific Trust considering its adoption. Such individualized assessments will not only allow each Trust to see whether the technology performs well within their systems and for their patients, but also contribute to the overall strength of evidence behind the technology. Building on this, one of the next steps would be to trial the effect of the technology directly on patient care. Ideally, this would take the form of a randomized controlled trial comparing groups who had CBT involved in their treatment pathway and those who did not.

Another key step to galvanize clinical adoption of CBTs within pathology is NICE approval. NICE produces technology appraisal guidance where the recommendations are based on “a review of clinical evidence and cost effectiveness” [26]. The manual for NICE health technology evaluations is extensive and states that “NICE considers all types of evidence in its evaluations” [27]. The authors of this manual explain that for “diagnostic technologies,” “end-to-end studies” are preferred, and “diagnostic accuracy studies” are also included in this document. A study such as ours could help provide essential information for these health technology evaluations and inform NICE recommendations.

As mentioned, for recommended technologies, NICE assesses information regarding cost-effectiveness, where it is asked, “does it represent value for money?” [26]. Its manual details a specified “reference case” that defines the methods for economic evaluation that NICE prefers [27]. Within this, they include cost-utility and cost-comparison analyses. To this end, a health economics evaluation in line with our study protocol has been considered. This evaluation would directly compare the cost of processing breast biopsies for ER, PR, and HER2 testing using the current standard of care (ie, immunohistochemistry with or without ISH) versus replacing it with a CBT (ie, without immunohistochemistry with or without ISH). To determine the resources used by the current diagnostic pathway within the LTHT laboratory, estimates will be taken directly from the existing standard of care used. This will include resources used for slide preparation; pathologist slide review and reporting time; costs of laboratory staffing, reagents, and equipment; costs of ancillary investigations for ER, PR, and HER2; and the costs of physical and electronic slide storage. These would then be directly compared to the costs of the CBT. This would include the time stamps collected in this study, covering the time taken by the CBT to analyze images and produce results, and the costs to the NHS of accessing the CBT reports. The estimation of the potential savings from the use of the CBT will include both straightforward and more complicated analyses. For directly comparable parameters, such as the cost of testing, the difference would be estimated. For parameters such as the potential savings of freeing up pathologists’ time in slide review for other uses, modeling will be used to estimate these scenarios. The wealth of information this evaluation could bring will again help inform the Trust of the CBT’s usefulness as well as provide evidence to NICE for a technology appraisal.

Finally, it would be beneficial to align a service evaluation such as ours with a degree of usability testing. It has been found from questioning pathology consultants in the United Kingdom that for acceptance of an AI tool, “high levels of usability” are key [28]. Moreover, King et al [28] found that their results “suggested an iterative design process with early user involvement” would help promote “AI uptake”. Usability testing is planned to accompany this service evaluation design. Separately, pathology trainees and consultants will be able to view and interact with the CBT, using it as intended but not on ‘live cases’. This will give the users a chance to explore the technology without any concern of error or interruption of normal care. Feedback from this testing will be gathered and used to help perfect the interface of the CBT.

### Limitations

As discussed by Colling et al [19] in their clinical practice road map, there are inherent issues with establishing a ground truth for some AI technologies. They describe that using a pathologist’s microscopic assessment as the ground truth is a “controversial assumption”. This is due to the interobserver variability seen and the subjective nature of the assessments. They suggest that validation is normally needed across multiple pathologists and laboratories. As our study design mimics the standard clinical pathway, it will solely rely on pathologists’ assessment of the case for ER, PR, and HER2 status. While this does not necessarily mitigate the issues highlighted, it would compromise the aim of this study to assess the CBT’s utility in a real-life clinical environment. To address this limitation, it may be necessary to carry out additional testing in a specific investigation. Additionally, as mentioned earlier, this protocol may not precisely fit within the infrastructure of other NHS Trusts. This lack of complete generalizability is unavoidable and is not wholly a limitation, as others can adapt this protocol to fit the needs of their departments, pathologists, and patients. While this protocol can be adapted to other Trusts, it is important to note that it does not assess the integration of a technology into a specific workflow. This has been deliberate, given that the focus of this design was to mitigate the premature integration of a newly developed and yet clinically unproven diagnostic technology. From a broader perspective, the foundations of this service evaluation could be implemented in health care systems outside the UK. With that said, as is the case for other United Kingdom sites, there will be logistical, regulatory, and clinical hurdles specific to each individual administration. For example, the NDOS appears to be NHS England specific [20] and would likely not need to be considered internationally. How the evaluation’s foundations may apply in other international diagnostic histopathology services is beyond the scope of this paper.

### Conclusions

The use of CBTs and AI within pathology in the United Kingdom is increasingly imminent. To validate and determine which technologies will perform best within a real-life clinical environment, clinical service evaluations, such as the one we have designed, are warranted. This protocol can provide a blueprint for other researchers and other pathology departments to enable their own evaluations. It may also provide the basis for further work needed, such as health economics evaluations and usability testing.

## Appendix

Multimedia Appendix 1 Funding confirmation.

Multimedia Appendix 2 Peer-review report from Innovate UK (IUK) Investor Partnerships SME Grant Committee.

## Abbreviations

AI: artificial intelligence
BC: breast cancer
CBT: computer-based technology
ER: estrogen receptor
H&E: hematoxylin and eosin
HER2: human epidermal growth factor receptor-2
ISH: in situ hybridization
LIMS: laboratory information management system
LTHT: Leeds Teaching Hospitals National Health Service Trust
NDOS: National Data Opt-Out Scheme
NHS: National Health Service
NICE: National Institute for Health and Care Excellence
PR: progesterone receptor
R-DIT: Research Data and Informatics Team
UKCA: UK Conformity Assessed
WSI: whole slide image

## Data Availability

Data sharing is not applicable to this paper as no datasets were generated or analyzed during this study. However, the authors plan to make the data of the study available as a follow-up publication, which will incorporate the principal findings of this clinical evaluation.
