# Supplementary material for: Deep-Learning Solution Providing Molecular Marker Subtyping of Breast Cancer Whole Slide Images: Protocol for a UK Clinical Service Evaluation Study
Source: JMIR Res Protoc. 2026 Jun 16;15:e76785. doi: 10.2196/76785 (PMC13320011; doi:10.2196/76785)
Supplement: Multimedia Appendix 1 [file resprot_v15i1e76785_app1.pdf]

Gmail

Naren Kumar <naren@panakeia.ai>

Important message about your application 'Multi-site prospective evaluation of AI-driven tool to accelerate Breast cancer diagnosis and treatment ' for the competition 'Innovate UK Investor Partnerships: SME round 6'

Innovation Funding Service <noresponse@innovateuk.gov.uk>
To: naren@panakeia.ai, noreply-ifs@innovateuk.org 22 April 2024 at 11:19

GOV.UK

22 April 2024 Innovate UK

Dear Narender Kumar,

Innovate UK

Innovate UK Investor Partnerships: SME round 6

Multi-site prospective evaluation of AI-driven tool to accelerate Breast cancer diagnosis and treatment

We are pleased to inform you that your application for Innovate UK Investor Partnerships: SME round 6 has been successful.

What to do next

We need additional details and documents before you can start your project. You must:

1. Sign into your Innovation Funding Service (IFS) dashboard

1. Complete the Project team, Bank details and Project details sections within 5 days of this email by 29 April 2024

We may contact you to clarify the project finances. This may include the financial viability and capability of your organisation. This process is managed through your IFS dashboard.

Bank details

The bank account the grant is to be paid into must:

- be a business account in the same name as the organisation listed in IFS
- be from a UK bank regulated by the Prudential Regulation Authority (PRA)

- have a cheque and credit clearing facility

Online accounts are eligible provided they meet the above criteria.

Innovate UK will accept most banking societies apart from:

- Viva Wallet
- Intesa Sanpaolo
- Equals Money UK Limited

There are accounts we deem "Non-standard" and will need additional checks which can delay the approval process, they are:

- Ziglu Ltd
- Air Wallex

We will contact you with queries using your IFS dashboard, which notify you by email. You must answer these promptly.

Document approval

As part of Innovate UK's commitment to continuous improvement, we are trialling an alternative approach to project setup - specifically the approval of your Spend Profile and Exploitation Plan.

We will contact you separately with more information about this in due course.

If you have any queries whilst completing project setup, email us atprojectsetup@iuk.ukri.org.

Starting your project

Once complete, Innovate UK will issue your Grant Offer Letter (GOL). The GOL is your formal contract with Innovate UK. The GOL must be signed, uploaded to your IFS dashboard and approved before your project is able to start. This includes an additional annex, which your investor partner signs and completes to confirm the investment. You must not start your project until your signed grant offer letter has been approved on IFS, we have confirmed the aligned investment meets the requirements and you have received confirmation that you can start your project.

You must make sure all documentation and queries are complete and your GOL has been approved within 90 days of this letter.

If you do not start your project within 90 days of this letter, funding may be withdrawn.

Monitoring your project

We will shortly begin assigning your project a Monitoring Officer.

How are successful projects monitored?

Assessment and Feedback

Your application has been assessed by independent assessors selected from industry and academia.

We received 72 submitted applications for this competition and 64 were sent for assessment.

This competition has applied a portfolio approach to ensure funds are allocated across strategic areas identified in the scope of the competition.

Publicising your Project

We have provided you with this information as we know it will be important for you to commence your project as soon as possible, however we ask that you treat this information in confidence at present and refrain from any media activity based on your application. As a public body, Innovate UK is subject to publicity restrictions during the sensitive period immediately before the local election. As a result of this, we cannot publicise the results of competitions as we usually would and ask that you also restrict any promotion of your success in this competition during this period. There are no restrictions on communications with your investor partner, but proactive PR should be avoided. If you have any queries, please contact: pressoffice@innovateuk.gov.uk

Innovate UK Business Growth

Innovate UK Business Growth will be notified of your success. Innovate UK Business Growth is a key part of Innovate UK's investment in the innovative businesses that drive UK economic growth, offering free bespoke business support to help businesses grow and scale. If you are an innovative UK registered micro, small or medium sized business (SME), you could be eligible for this support. Visit the Innovate UK Business Growth website to find out more.

There are No Limits to your potential. Please visit No Limits to search for relevant opportunities and other pathways to success in innovation offered by partner organisations — from skills and training to investment and advice.

Interested in turning your ideas into reality? The innovation hub has public funding and support for innovation in one place. Please visit the innovation hub to start

searching for support and funding.

Contact us

If you have any queries whilst completing your project set up email us at projectsetup@iuk.ukri.org.

You can also watch our video What steps are there before a project starts? - YouTube

N.B Please remember comments relating to Document Approval will differ for this specific project.

Congratulations on the success of your application.

Yours sincerely,

Innovate UK, part of UK Research and Innovation

Tel: 0300 321 4357

Email: support@iuk.ukri.org

Innovate UK, part of UK Research and Innovation
Polaris House
North Star Avenue
Swindon
Wiltshire
SN2 1FL

© Crown copyright

This email and any attachments are intended solely for the use of the named recipients. If you are not the intended recipient you must not use, disclose, copy or distribute this email or any of its attachments and should notify the sender immediately and delete this email from your system. UK Research and Innovation (UKRI) has taken every reasonable precaution to minimise risk of this email or any attachments containing viruses or malware but the recipient should carry out its own virus and malware checks before opening the attachments. UKRI does not accept any liability for any losses or damages which the recipient may sustain due to presence of any viruses.
