# Supplementary material for: Deep-Learning Solution Providing Molecular Marker Subtyping of Breast Cancer Whole Slide Images: Protocol for a UK Clinical Service Evaluation Study
Source: JMIR Res Protoc. 2026 Jun 16;15:e76785. doi: 10.2196/76785 (PMC13320011; doi:10.2196/76785)
Supplement: Multimedia Appendix 2 [file resprot_v15i1e76785_app2.pdf]

The Innovate UK office will be closed from 5:00pm on Friday 2 May until 9:00am on Tuesday 6 May 2025. Please email support@iuk.ukri.org and we will reply when our phone lines reopen.

# Project details

## Subsidy basis

| Partner | Funding rules | |
|---|---|---|
| PANAKEIA TECHNOLOGIES LIMITED (Lead) | Subsidy control | View answers |

## Application team

## PANAKEIA TECHNOLOGIES LIMITED

Organisation details

| | |
|---|---|
| Type | Business |

### Team members

| Full name | Email |
|---|---|
| Pahini Pandya | pahini@panakeia.ai |
| Narender Kumar | naren@panakeia.ai |

## Application details

**Competition name**
Innovate UK Investor Partnerships: SME round 6

**Application name**
Multi-site prospective evaluation of AI-driven tool to accelerate Breast cancer diagnosis and treatment

**When do you wish to start your project?**
15 May 2024

**Project duration in months**
18 months

**Innovation area**
Precision medicine

**Has this application been previously submitted to Innovate UK?**
No

## Research category

**Selected research category**
Experimental development

## Equality, diversity and inclusion

No feedback provided

**Have you completed the EDI survey?**

Yes

## Project summary

No feedback provided

**Project summary**

This **18-month project** on advancing PANProfiler Breast (PPB), an innovative breast cancer diagnostic product, **from Technology Readiness Level 8 (TRL8) to TRL9**, **aligns with the Health and Wellbeing scope of the IUK Investor SME Partnerships call.**

**Seeking Innovate UK support to match VC investor funding (commitments secured)** as a part of current raise, Panakeia aims to evaluate PPB in real-word settings for diagnosing ER, PR, and HER2 status directly from images of H&E stained biopsies/resections.

Panakeia's approach employs Machine Learning (ML) for rapid and accurate identification of morphologic patterns in H&E-stained tissue images indicative of ER, PR, and HER2 status. **This innovative solution stands out as the current gold standard lab-tests**:

- require specialised instrumentation for immunohistochemistry (IHC) and in-situ hybridization (ISH).
- take 3-15 days for results
- cost ~£450/patient (NICE)
- require pathologist scoring for IHC/ISH which has low accuracy and high discordance (<70%, Fernandez AI, JAMAOncology2023)

Incorporating AI/ML solutions to improve IHC-scoring accuracy adds more steps, with low accuracy improvements (43 -75%).

**Key Objectives:**

**1. Multi-Centered Prospective Study:**

- Execute a prospective study on 800+ cases across 2-3 clinical sites, leveraging those already involved in the retrospective evaluation.
- Generate robust evidence to elevate PPB to a standard suitable for clinical adoption.
- Expand PPB adoption within pathology teams at the local sites and build buy-in from the oncology multidisciplinary team.

**2. Budget Impact Models and Health Economics Evidence:**

- Develop budget impact models illustrating time and cost savings, *crucial* for clinical and commercial adoption.
- Generate health economic evidence for subsequent Health Technology Assessment (HTA) supporting NICE recommendation.

**Project Outputs/Deliverables**

**Clinical Validation and Safety Assessment:**

- Demonstrate comparable performance to existing gold-standard tests for PPB in a prospective setting across 2-3 clinical sites.
- Submit reports, abstracts for conferences, and manuscripts for peer-review publication.

**Clinical Team Onboarding and Procurement Planning:**

- Onboard more than one pathologist at each participating site to PPB
- Develop a plan with a clear route to procurement post-project with partner NHS sites.

**Budget Impact Models and Health Economic Model:**

- Provide budget impact models for each hospital site, quantifying the 1-year budget impact.
- Develop a health economic model for subsequent HTA assessment for NICE recommendation.

This endeavour **builds on Panakeia's existing capabilities** and **facilitates rapid commercialization post-project within the NHS**, starting with project subcontractors (existing customers). Successful completion of this project is anticipated to expedite NHS adoption and pave the way for global commercialization through partnership deals with big pharma (in the pipeline).

## Public description

No feedback provided

**Public description**

This project focuses on real-world testing of an AI-tool which informs the diagnosis and treatment of breast cancers in minutes, without the need for wet lab based tests.

Currently, treatment decision-making for cancers requires several molecular tests subsequent to the confirmatory diagnosis of cancer by a pathologist. With increasing breast cancer incidence, increasing number of wet-lab test and an ageing pathologist workforce, there is a need for solutions capable of relieving workload pressures in a cost- and time-effective way.

The 18-month project, which has the firm backing of a specialist patient advocacy group, aims to evaluate Panakeia's diagnostic solution to aid NHS breast cancer services by reducing pathology workloads, streamlining diagnostic workflows, shortening turnaround times and reducing costs.

## Scope

In scope 5/5

**How does your project align with the scope of this competition?**

This **18-month Panakeia-led project, costing £1.273M and seeking £572,720 (~45%) in IUK grant funding**, is well within the scope of this competition as it:

- Involves **experimental development**, evaluating the real-world performance and health economic impact of Panakeia's innovative digital, data-driven tool for Breast Cancer for determining ER, PR & HER2 biomarker status from H&E biopsy images (from TRL 8 to TRL9) across 2-3 clinical sites
- **SME-Led**
- **able to complete the aligned investment** led by the investor partner. Commitments for ~£700k already secured (Ref-Qu4)

- Directly **aligns to the "Health and wellbeing" theme** of the call via focus areas falling under Biomedical Catalyst as well as "Equality, Diversity and Inclusion" area (Refer-Qu3)
- Indirectly supports "Net Zero" and "Next generation digital technologies" focus areas of this call
- The project will **enable clinical adoption and rapid commercialisation of Panakeia's solution immediately post-project** as it builds on retrospective evaluation of PPB by existing clinical partners. This project will generate the final piece of real-world evidence needed by these NHS hospitals to adopt the solution. This will
    - **Deliver significant market impact (ROI of 1,257%)** via generation of 150 jobs in next 5 years, \>£16Mn 5-year cumulative revenue for PPB and saving the NHS £31 million over 5-years
    - **Enable scalability**:
    - Scaling Panakeia's solution adoption within NHS by paving the way for other products in current portfolio
    - Help accelerate international adoption via generating evidence for hospital sites & big pharma partnerships already in the pipeline (Ref-Qu9)
    - Creating learnings that can be expanded to new product development
- Will **help Panakeia attract additional equity investors** (already in pipeline) by achieving the milestones needed to raise a series A in 18 months (Ref-Qu4)
- Is **led by an experienced leadership team** working with clinical partners having a track record of delivering new-diagnostic solutions for cancer within NHS & globally (Ref-Qu7)
    - The team has raised \>£4.5M till date from Individuals, Family Offices and VC funds
    - Raised \>£0.5M in grants including IUK funding
    - And has already delivered 2 UKCA/CE-marked products leveraging their technology

### Assessor feedback

**Assessor 1**

The applicants intend to test their innovation in a RWE study

**Assessor 2**

The proposal meets the eligibility criteria and the work proposed is well within the remit of the call.

**Assessor 3**

A UK SME delivering a potential next generation Ai assisted solution (TRL7/8 to 9) for Breast Cancer determining key biomarker status as disease pathology markers and for care pathway management.

**Assessor 4**

In scope.

**Assessor 5**

Panakeia is a UK based SME with an automated machine learning based approach to diagnosing great cancer from H&E biopsy images addressing the health and well being, and precision medicine interests of Innovate UK. This is an 18 month industrial research clinical validation study to take the project from TRL8 to TRL 9

# Application questions

## 1. Applicant location (not scored)

No feedback provided

### Applicant location

Panakeia Technologies Limited.

Registered Address:

Salisbury House,

Station Road,

Cambridge

CB1 2LA

Confirmed Subcontractors for the project include Leeds University, Leeds Teaching Hospitals NHS Trusts and NHS Lothian all located within the UK.

Back-up subcontractors include: Sheffield Teaching Hospitals NHS Trust, NHS GGC, Royal Marsden NHS Trust, University Hospitals Coventry and Warwickshire, Addenbrookes NHS Trust, Velindre NHS Trusts - all located within the UK as well.

## 2. Animal Testing (not scored)

No feedback provided

### Animal Testing

No

## 3. Specific theme (not scored)

No feedback provided

### Specific theme

This **18-month project** focuses on real-world evaluation and health economic assessment of Panakeia's **innovative digital, data-driven tool** for Breast Cancer for determining ER, PR & HER2 biomarker status from H&E biopsy images. This solution enables:

- **Faster, more accurate diagnosis** and

- **Precision medicine via improved patient stratification** for treatment decision-making

This 18 month project is **aligned with "Health and wellbeing" theme** of the call specifically because:

- Precision Medicine, Health Technology (Diagnostics, Medical Technology and devices) and Digital Health Technology (Data driven health, AI in health) areas of focus for Biomedical Catalyst
- Next generation immunotherapies and clinical decision support tools to optimise therapeutic dosing areas of focus for Transforming Cancer Therapeutics

Furthermore, **Panakeia's solution also supports goals of additional themes** within this call including:

- **Equality, Diversity and Inclusion**
  - As this is an innovation developed by a team of diverse talent from underrepresented groups including:
  - BAME female founder & CEO
  - Wider leadership team (excluding CEO) of 5 individuals all from BAME backgrounds with 3:2 (M:F) split across genders
  - The team has established new role models in AI, Healthcare and the VC-backed startup communities (Refer-Qu7).
  - This innovation focuses on improving patient outcomes especially those in the socio-economically deprived groups which face longest wait times for cancer (Refer-Qu10)

- **Net Zero**:
  - Panakeia's solution reduces Carbon emissions by ~3.6x by lowering the need for specialised lab equipment, electricity usage and transport of samples (via fewer lab tests). Refer-Qu10

- **Next generation digital technologies**
  - By using AI to boost NHS productivity

## 4. Investor partner (not scored)

No feedback provided

**Provide the name of the investor partner who you are working with.**

**Investor Partner: Ascension Ventures Limited** (Partner: Chris Wheatcroft)

Panakeia is **currently raising ~£1.273 Mn in new investment** for a 18 month project to:

- Deploy PANProfiler Breast (PPB) in 2-3 NHS sites (sites listed in Qu7)

- Generate real-world clinical and health economic evidence needed for clinical adoption & NICE recommendation

**Panakeia already has secured VC commitments totaling ~£700k (55%)** from several investors already including insiders such as: Ascension Ventures Limited, Roger Ferguson Jr, Hoxton Ventures as well as new investors including Eastbourne partners and Modi Ventures.

T**he company is seeking £572,720(45%) in match funding from Innovate UK** for this experimental research project.

The hospital deployment achieved as a part of this funding will allow Panakeia to close in additional investors in the pipeline in order to subsequently scale the clinical adoption of it's PPB product post-project.

## 5. Need or challenge

Average score 8.6 / 10

**What is the business need, technological challenge, or market opportunity behind your innovation?**

**Motivation:**

**1 in 7 women face breast cancer, yet only 69.5% meet faster diagnosis standards, with 68.5% accessing treatment within 2 months** (NHS National Data, June 2022-Jan 2023). Timely detection is vital, as each month's delay results in a 10% drop in survival rates. Pathology, responsible for 41% of delays, grapples with a shrinking workforce and heightened wet-lab test demands (RCPath & NHS stats). Addressing these challenges is crucial for improved outcomes in breast cancer patients.

**Challenge/Opportunity:**

**BC diagnosis and treatment relies on ER/PR/Her2 molecular tests** post-pathologist identification of cancer using H&E-stained biopsies. ER/PR/Her2 status is determined by IHC, requiring additional Her2 testing via ISH in 25-40% of cases. **Turn-around times range from 3-5 days for ER/PR to 7-14 days for HER2**, contingent on in-house or outsourced testing (with approximately 70% Her2 testing outsourced).

**Similar Innovation Limitations:**

Current methods for **ER, PR, and Her2 testing** involve specialised wet-lab tests (IHC/ISH) with prolonged turnaround times and **costing ~£450/patient (NICE)**. Existing AI-based solutions for IHC quantification add complexities to workflows and incur additional costs. **Panakeia's innovation addresses these limitations** by offering a comprehensive solution that eliminates the need for IHC/ISH tests, thus providing faster, cost-effective, and accurate results. While there are ML

approaches for H&E-based profiling published in literature, they have had limited performance. Panakeia's PPB is the ***world's first and only*** UKCA/CE-marked offering for ER,PR,HER2 on the market till date (***enabled by 10+ years of proprietary know-how and patent-pending technological breakthroughs***). For further details, refer AppendixQu6.

**Prior Work:**

**PPB has been retrospectively validated across 5 NHS sites and achieved an average accuracy of 96.74% (±1.1%), 95.5% (±1.5%) and 92.2%(±8.4%) for HER2, ER and PR, on 2000+ retrospective cases** (posters- ESMO (https://shorturl.at/tBR07): https://shorturl.at/tBR07, SABCS (https://shorturl.at/fhkuE);PO3-07-05,p1893: https://shorturl.at/fhkuE). This project expands Panakeia's existing capabilities to bring a TRL8 stage product to TRL9 via validating PPB's performance in a real world clinical setting for accuracy, reduced TAT, hospital/trust cost savings and health economic impact for the NHS.

**Wider Influences:**

Rising BC rates and a diminishing pathology workforce underscore the need for advanced diagnostics. **NHS long-term plan and government investments in AI, precision medicine, early detection and digital pathology provide an opportune environment. Our project aligns with these initiatives**, offering a comprehensive solution for improved BC diagnosis and treatment.

## Assessor feedback

### Assessor 1

The applicants state that reducing the time to receive biomarker results and improving diagnostic capacity are key needs. The innovation would be considerably faster than alternatives, and could fundamentally change patient workflows, so in this regard there would be an important need in the market for this solution. Equally, the potentially higher levels of accuracy and redundancy from alternative solutions mean that there are other competitors in the market.

### Assessor 2

Excellent motivation for the project with specific healthcare needs well identified and quantified. Wider factors including healthcare resources have been discussed and are specifically addressed by the proposed new approach. Some discussion on the technological challenges as identified from the prior work would have strengthen the proposal.

**Assessor 3**

Good account, lacking detailed supporting references. Outline good. It would have been impactful to understand why NHS validation did not unlock Series A funding for ongoing work.

**Assessor 4**

There is a good motivation for the project. There is a good awareness of the nearest innovation and some understanding of limitations and any similar innovations.

**Assessor 5**

The applicants have provided a detailed breakdown and assessment of the clinical need for better faster diagnosis of breast cancer and specifically ER, PR and HER2 based disease. The current challenges in terms of resource , time and accuracy are discussed as are existing clinical diagnostic pathway and competitor approaches. The application of Panakeia exisiting technology to address the challenges are discussed in terms of addressing NHS long term initiatives

## 6. Approach and innovation

Average score 8.6 / 10

**What approach will you take and where will the focus of the innovation be?**

**Innovation, improvement, product offerings, FtO & outputs details in AppendixQu6.**

**Response:**

Panakeia tackles challenges (**Refer-Qu5**) with PPB, utilising ML to rapidly determine ER,PR,HER2 status from H&E tissue images, offering results in minutes at 1/3rd the cost (**Refer-Qu9**). The ML-models correlate morphologic patterns in H&E-stained tissue with molecular changes. This project aims to evaluate PPB, a UKCA/CE-marked product, in prospective clinical settings (from TRL8 to TRL9) to generate real-world evidence of clinical performance, health economic evidence to support NHS adoption.

**State-Of-Art Improvement:**

Panakeias' patent-pending technology offers significant competitive advantage over market alternatives. Developed for clinical use, PPB boasts a **significant speed advantage** over State-of-the-art wet-lab tests, is already CE/UKCA marked and can be readily deployed, immediately post-project, to Panakeia's existing clinical partners/customers **without infrastructure changes**. This **pioneering approach establishes a new market category (AI to replace wet-lab tests)**, differentiating from competitors who:

● Develop wet laboratory molecular tests (the existing standard-of-care) such as immunohistochemistry (Roche) and genetic tests (Qiagen, Promega, etc.).

● Develop AI-solutions to replicate pathologist decision-making for determining if a tissue sample is normal or abnormal (Proscia), cancer detection (Paige, Ibex,) and analysing/scoring immunohistochemistry assay results (Visiopharm/Mindpeak).

**Innovation Focus:**

PPB leverages Panakeia's novel technology to offer ER/PR/HER2 status for Breast cancers in minutes. This **project focuses on development of new solutions for existing areas** and represents the first-of-its-kind UKCA/CE-marked solution for Breast cancer testing.

**Freedom-To-Operate (FtO):**

Panakeia has **filed 3-patents covering North America, Europe, and Asia**. A **thorough FtO analysis across various patent databases ensures operational freedom**. With confidence that existing patents do not hinder operations, Panakeia maintains regular vigilance with its IP counsel, Marks & Clerk LLP.

**Project-Fit:**

**This project strategically aligns with Panakeia's portfolio** by addressing current customer demands through delivering the final piece of clinical & economic evidence needed for clinical adoption. This project will **also prime NHS sites to adopt Panakeia's 2nd UKCA/CE-marked product** for colon cancers (currently undergoing IUK-supported multi-site retrospective evaluation). The project also **positions Panakeia for establishing big-pharma partnerships** around companion diagnostic development and global expansion.

**Competitive-Gains:**

The project **enhances competitiveness by accelerating clinical and commercial adoption of a world-first technology** to address unmet NHS needs in UK. The **know-how generated during the project can be leveraged across other product lines**. Furthermore, the project **opens avenues for strategic partnerships**, particularly with major pharmaceutical companies, facilitating accelerated global commercialisation establishing Panakeia (UK-based company) as a leader in the space.

[Appendix Qu6.docx.pdf (opens in a new window) (/application/10114168/form/question/39391/forminput/108585/file/672964/download)](/application/10114168/form/question/39391/forminput/108585/file/672964/download).

## Assessor feedback

### Assessor 1

This approach is highly innovative and at the forefront of AI-assisted diagnostics. There are substantial opportunities to scale both this innovation and to apply this technology to other biomarkers or therapy areas.

### Assessor 2

The proposed approach is well aligned with the needs identified in Q5\. The approach is innovative as despite the large amount of AI/ML resources available, few have made the clinical translation so the proposed solution could provide a step-change in breast cancer diagnostics. Interesting mention of application to colon cancer but the link with big-pharma is less clear. The technical approach has been well described and the use of AI/ML is well justified. Comparison with similar approach would have been beneficial. Again little information on the technical challenges has been provided. Integration into the clinical flow has been carefully considered.

### Assessor 3

Solid empirical evaluation impacting current care pathways. A good competitor market overview by technology and value statement using the new approach.

### Assessor 4

The approach addresses the need or challenge, and the main innovations are highlighted. Evidence shows that the proposed development is innovative, and that the applicant has the freedom to operate.

### Assessor 5

The applicants have described the current diagnosis pathway and the performance of existing competitor approaches is described with comparator to the panakeia process. The provided evidence does demonstrate potential time saving in the process. It is unclear how long the slide preparation will take as it is not demonstrated int he other existing pathway. The applicants hold

several patents and have conducted extensive searches to determine they have freedom to operate

## 7. Team and resources

Average score 8.2 / 10

**Who is in the project team and what are their roles?**

**Details on team, roles, skills, experience, resources/facilities, subcontractors and back-up subcontractors and roles needing hiring in AppendixQu7.**

**Founded on the CEO's patient experience**, Panakeia provides ML-driven solutions for molecular biomarker profiling directly from H&E stained tissue images. The company is backed by notable partnerships with leading cancer centres, hospitals & universities. Leadership team includes:

**Dr. Pahini Pandya (CEO,founder)**, holds a decade-long experience in translational cancer research, with a PhD from KCL and post-doc from Cambridge. In addition, she has business experience from Stanford GSB and Cambridge JBS, where she helped over 25 UK start-ups commercialise their research. Role: Project Lead & Lead WP1,8

**Dr. Narender Kumar (Program&partnerships manager),** has a decade of experience in translational research with a PhD in genomics. He's led multiple large initiatives for clinical adoption of genomics for diagnostic applications in multiple countries.Role: Project Manager, Lead WP2,7; Involved in WP1,6,8

**Dr. Salim Arslan (Research Lead)**, leading expert with 14+ years experience in ML research (industry & academia) with multiple publications at top ML and biomedical imaging conferences/journals including MICCAI, IEEE TMI; Honorary Research Fellow at Imperial College London. Led development of core proprietary, patent-pending ML technology for Panakeia's products and platform. (Refer-AppendixQu6).Role: ML Lead, Lead WP3,6; Involved WP1,2,5,8

**Dr Andre Geraldes (Tech Lead)**, PhD in Computer Science with 12+ years in research and engineering, published several journals/conferences including IEEE and Frontiers in Robotics and AI. Extensive experience in safety-critical fields like ML and surgical robotics. Led development of Panakeia's scalable infrastructure, enabling rapid capacity scaling and new product rollout. Role: Lead WP4,5; Involved WP1,3,2,6,8

**Angelica Marcia (Regulatory Lead)**, has successfully led Panakeia's efforts in gaining UKCA and CE-marks for their existing portfolio products with 4+ years experience in regulatory space (Refer-AppendixQu6). Role: Involved WP8

**Dr Shikha Singhal (Lead Pathologist)**, Consultant NHS Pathologist with 10+ years experience in leading Pathology teams, clinical research and a special interest in GI & Breast cancers.Role: Involved WP2,6,8.

Wider team involves 3 ML scientists, 1 pathologist & 1 software engineer.

**Panakeia will hire a Software Engineer and Chief of Staff/Head of Operations**.

**Confirmed subcontractors** for this project include **Leeds Teaching Hospitals & NHS Lothian as clinical sites** as well as **University of Leeds for Health Economic Evaluation**. Panakeia has worked with both NHS sites for retrospective validation of PPB and has worked with University Leeds on various projects since 2020. **Details and Back-up sites in AppendixQu7.**

Appendix Qu7.docx.pdf (opens in a new window) (/application/10114168/form/question/39392/forminput/108591/file/672947/download).

### Assessor feedback

**Assessor 1**

There is an excellent team with a broad range of specialist skills required for this project. There is good evidence of traction in the market.

**Assessor 2**

The team is perfectly placed to deliver the proposed work. The leading members of the team have considerable expertise in relevant fields including AI/ML, pathology, healthcare translation and regulatory approval. Their role within the project has been clearly identified although there appears to be some considerable overlap for ML development and pathology. Additional and more specific details about previous activities would have been beneficial to support the level of expertise. Strong support from subcontractors

**Assessor 3**

A solid team well within their capabilities to take the project to a successful conclusion.

**Assessor 4**

Good strong project team with relevant experience. Need stronger commercial experience.

**Assessor 5**

The project team have been identified and project role clearly stated with relevant prior experience detailed for the key individuals. The applicants state they will hire a software engineer (justifiable for the project) however the need for a Head of operations in relation to this project is not clear The three named subcontractors will provide the necessary resources for the clinical evaluation of this project

## 8. Market awareness

Average score 8.6 / 10

**What does the market or markets you are targeting look like?**

**Target Market & Size:**

Panakeia's PPB primarily targets the global BC diagnostics market, **valued at £17.02 billion in 2022** with an estimated £22 billion by 2029 (CAGR-4.3%). **Initial emphasis will be on the UK**, where the annual cost of ER,PR,Her2 testing is approximately £25.2 million, serving ~55,920 new cases yearly.

**Market Dynamics:**

The main **customer segment** is NHS hospitals utilising digital pathology (DP) for breast cancer diagnosis. **Anticipated growth from 20% to 80% of trusts within five years** establishes a **serviceable addressable market (SAM) of ~£20.2 million**. The project helps the NHS meet cancer wait times (FDS & 62-day to treatment) by addressing long laboratory turnaround-times, and responds to increasing BC incidence, pathologist shortages, and **builds on UK government/Innovate UK investments in Genomics, DP, Precision Medicine & AI.**

**Supply/Value Chains:**

Panakeia supports multiple image formats (Philips, Leica, Olympus, Roche, Hamamatsu etc.) and integrates with Picture Archiving and Communication Systems (PACS) & Laboratory Information Management Systems (LIMS) providers like Sectra. This facilitates seamless integration into existing diagnostic workflows within NHS pathology networks.

**Business Models:**

Panakeia integrates its software through PACS/LIMS software, offering **volume-tiered software licences to pathology networks at £50/biomarker/test**. This pricing is competitive, positioning Panakeia as a cost-effective alternative to traditional IHC tests (£150/IHC/biomarker).

**Barriers and Drivers:**

**Barriers**, including clinician acceptance, integration challenges, new market entry, pricing acceptability, and regulatory approval timelines, are mitigated through established customer relationships, product integration, grant funding, pharma partnerships, and competitive pricing. **Adoption drivers** encompass faster laboratory turnaround times, increasing BC incidence, pathologist shortages, changes in Her2 treatment, and government investment in digitisation of NHS pathology.

**UK Position:**

The **project aligns with the UK's leadership in DP, AI, and precision medicine, building on past IUK-funded initiatives**. It supports national strategies, including the Life Sciences Vision, Genome UK, data saves lives, NHS Long Term Plan, and Faster Diagnosis Standard, enhancing the UK's global standing in healthcare innovation.

**Unexplored Markets (& size):**

Panakeia's technology, effective in 32 cancers (including BC), opens new markets for precision medicine. The estimated market-size for BC alone in unexplored markets is ~£2.9 billion, based on 14.7 million new cases at £200/assay(average) in line with existing commercial opportunities.

**Exploring Unexplored:**

Project success will catalyse big pharma partnerships (incl. Astrazeneca & Novartis; Refer-AppendixQu7), allowing Panakeia to expand its product portfolio into new markets, disease areas, and biomarkers, solidifying its position as a leader in precision medicine.

## Assessor feedback

### Assessor 1

There is a very good understanding of the market size and the dynamics. There is an excellent serviceable market with several additional markets that could realistically be entered in the future.

### Assessor 2

Very good market analysis with relevant, specific and quantitative information provided. Some discussion on the current marker leaders and potential competitors should have been included. Target customers have been identified and quantified. The proposal also includes a proposition value which appears to be appealing and competitive. Potential barriers have been identified (although again, competition has not been discussed) and additional details on the mitigation strategies would have been beneficial. Good information on possible secondary markets.

**Assessor 3**

A strong and well thought through market statement. Details from earlier competitor landscape analysis and comparisons would have been helpful.

**Assessor 4**

There is a good awareness of the target market's drivers and dynamics. The market size is quantified with some evidence. For a new market, a good attempt is made at describing the possible routes to market and estimating the market size.

**Assessor 5**

The global diagnostic market and the addressable Breast cancer market is fully described and shows good understanding of the needs for market entry Potential barriers to entry and adoption are well described, the supply chain is well understood and the ability to use multiple imaging inputs and the PACs system shows universal applicability. Applicability to the NHS long term plan is clearly explained

## 9. Outcomes and route to market

Average score 8.4 / 10

**How are you going to grow your business and increase long term productivity as a result of the project?**

**Current Position:**

**Panakeia has just concluded several NHS pilots (including some funded by local ICB Refer-AppendixQu7)** for retrospective evaluation of PPB (world's first), integrated into their clinical workflows & software (e.g. Sectra). This project will

establish Panakeia's position in the pathology market with its existing UKCA-marked product, for identification of ER, PR, and Her2 biomarkers for BC.

**Target Customers/Value:**

Panakeia **targets NHS hospitals** using digital pathology for BC diagnosis (currently 20% of hospitals). **Panakeia's value proposition** centres on enhanced accuracy, reproducibility, workflow efficiencies, rapid turnaround times, and cost savings. Priced at £50/biomarker/test, it outperforms and offers significant cost reductions against current IHC/ISH tests (£150/£180; NICE benchmarks and FOI requests).

**Route to Market:**

PPB will initially be commissioned at Trust & ICB-levels. Trusts will procure volume-tiered (patient volume) PPB software licences. The project advances technology to TRL9, preparing for rapid commercialization post-project:

- **Year 1:** Commercial adoption (£150/patient/3 markers) with six NHS hospitals (including existing partners like LTHT, SYB, NHS GGC, NHS Lothian and Dr. Lal Labs (India); pipeline Refer-AppendixQu7); Secure additional VC investment and NICE accreditation. Establish big pharma partnerships for US+EU entry.
- **Year 2:** Expand across five NHS pathology networks in pipeline (incl. Peninsula Pathology network, North East London Cancer Alliance, Black Country Pathology Services). Scaling through channel partners and marketing (publications, conferences).
- **Year 3-5:** Nation-wide growth with existing PathLAKE, NPIC, iCAIRD partnerships post-NICE. CE-IVDR, FDA approvals. Develop new products for different cancers.
- **Year 4-5:** US+EU market-entry, targeting additional revenue ~£4.6M/annum, Pharma-partnerships for new products.

**Profit:**

Project outcomes will help Panakeia **secure big-pharma partnerships** post-project (ongoing conversations include AstraZeneca & Novartis; Refer-AppendixQu7), generating **£16M+ in cumulative revenue within five years**. Panakeia expects to serve ~44,700 UK breast cancer patients annually, yielding NHS cost savings of £31M and a **projected ROI of 1,257%**.

**Productivity/Growth:**

The project is expected to unlock additional VC funding, leading to the hiring of 3-4 new employees within the first year, ~40 within three years, and ~150 within five years, enhancing productivity and fostering sustained growth.

**Exploitation:**

Project's outcomes will be **disseminated through peer-reviewed publications**, contributing to Panakeia's clinical-evidence base. **New know-how** generated will be **leveraged for clinical adoption of other UKCA/CE-marked** products in Panakeia's portfolio following the project's conclusion.

**Expansion to Other Markets:**

Expansion will be **facilitated by** leveraging **pharma partnerships**, **existing channel partnerships** (e.g., Sectra), and collaborations with leading cancer centres. Utilising **FDA and CE marks** will expedite country-specific approvals, particularly in Asian markets.

## Assessor feedback

**Assessor 1**

The team describe an excellent route to market. While it is often useful to focus on a single customer initially, the team are able to address multiple customer segments because of the nature of their scalable technology. The NHS is notoriously difficult to enter; however, this approach is an exception because of the excitement around pathology-based innovations. There is a compelling business case, which strengthens the application.

**Assessor 2**

Very informative plan on the route to market. The timescale and expected revenue (adequately supported by relevant information, appear to be ambitious but feasible. Some discussion on the marketing plans and current position of the applicant in the market would have been beneficial. Scalability of the proposed solution to satisfy demand is not been specifically addressed although the big-pharma partnership may address this (clarity about this point would have been beneficial).

**Assessor 3**

A good commercial strategy that is detailed and ambitious but able to be delivered.

**Assessor 4**

Target customers are identified along with the value proposition to them. The routes to market and how profit, productivity and growth will increase is outlined with sufficient evidence.

**Assessor 5**

As a company nearing TRL 9 the route to market is very clear and is explained with planned growth of the company over 5 years and expected outcomes clearly justified The projected revenue over 5 years and anticipated ROI, if successful, is a credible expectation for the technology with the described route to market

## 10. Wider impacts

Average score 8.6 / 10

**What impact might this project have outside the project team?**

**Output:**

This project aims to advance Panakeia's ML-based molecular profiling technology for ER,PR and Her2, bringing it from TRL 8 to TRL 9, with a focus on the prospective clinical evaluation (2-3 sites). The anticipated outcome is a clinically-validated product **poised for clinical adoption & immediate commercialisation post-project**.

**Impact:**

**Customers:** Enables a 1-day turn-around time, supporting NHS trusts in meeting the 28-day FDS and the 62-day time to treatment. This will result in freeing up around ~600 biomedical scientist hours & ~9000 pathologist hours/ annum with cost savings of up to £6 million/annum in direct costs to the NHS through a reduction in IHC and ISH tests.

**UK Economy:** The project foresees £31 million in 5-year cumulative NHS cost savings and an impressive ROI of 1,257%. Additionally, job creation facilitated by VC funding and a big pharma partnership is expected, with plans for 3-4 new employees in the first year, ~40 within 3 years, and ~150 within 5 years.

**Additional Impacts:**

- **Alignment With Government Priorities:** including AI strategy, Data saves Lives, Life Sciences Vision, Genome-UK, NHS NetZero, and NHS Long Term Plan (cancer, digital transformation, personalised care, and workforce enhancements).

- **Environmental:** Panakeia's digital solution is expected to have **\>3.6x lower Carbon-emissions than traditional IHC**, with reductions in electricity, reagent usage, sample transport, and toxic chemical usage in wet-lab tests.
- **Regional & Socio-Economic:** The digital solution promotes equitable access to precision medicine, especially for patients in the most deprived quintiles (NICE-2021), through a highly accurate, user-friendly tool deployable in smallest pathology departments for rapid patient stratification.

**Social Impact:**

**Quality of life:** PPB's 1-day turnaround time can significantly improve patient survival rates, with an estimated 91% meeting 28-day FDS (3268 more patients) and 88% meeting 60-day time-to-treatment (4566 more patients) compared to current rates of 69.5% & 60.7% (NHS National Statistics).

**Social inclusion:** Cost reduction improves precision diagnosis accessibility across diverse socio-economic groups, especially in global markets.

**Jobs: PPB reduces risk of errors**, augments pathologists by identifying patterns in tissue invisible to the human eye, **simplifying Biomedical Scientist** tasks by transitioning from multi-step wet-lab assays to software-based analysis. Potential displacement of wet-lab assay providers are acknowledged challenges.

**Education:** The project generates educational know-how for AI integration in routine care, facilitating widespread dissemination across the NHS during PPB adoption scaling.

**Diversity:** To mitigate biases, the project ensures clinical evaluation on a statistically significant number of cases, including minorities, promoting equitable diagnostic outcomes.

## Assessor feedback

### Assessor 1

This company represents the forefront of how AI can be used to augment healthcare and democratise gold-standard provision.

### Assessor 2

Excellent potential socio-economical impact. The applicants have a clear understanding of the field and the market and a clear vision of the potential impact of the project. Relevant, specific and quantitative information have been provided on the expected impact which, although ambitious, are within reach of the project outcomes and the applicants. Additional (specific) information regarding EDI issues and mitigations would have been beneficial.

Potentially negative impact (mainly related to wider and unregulated/unsupervised use of AI/ML) should have been discussed.

**Assessor 3**

Good statement of impact and would have been enhanced by industry corroboration in support of details.

**Assessor 4**

There is good awareness of how the project may impact others outside of the team. Some evidence of social, economic and environmental impacts is considered.

**Assessor 5**

There is a clear description of expected benefits to healthcare providers (in terms of costs and resources) and the economic benefits to the UK economy. Additional impacts in terms of alignment to government priorities, and environmental benefits are described. The socioeconomic and regional benefits in terms of increasing accessibility in smaller hospitals and pathology departments Several social impacts are considered that have credibility. Negatives such as loss of jobs in wet labs have been considered although the displacement is likely to be absorbed to support other relevant assays

## 11. Project management

Average score 7.6 / 10

**How will you manage your project effectively?**

**Full work-plan, GANTT chart, deliverables/milestones (AppendixQu11).**

The **plan carefully considers resource requirements** of key innovative aspects in WPs2-6 **and is achievable within timescales and budget**, based on previous product deliveries.

**WP1: Project Management and Reporting** (Cost: £137,649; 288 days)

**WPL: Dr. Pahini Pandya (PP)**. Supported by program & partnerships manager (Dr. Narender Kumar,NK) and operations manager/chief-of-staff (to be hired), will

be responsible for managing progress, partners, decision making, budgeting and reporting.

**WP2: Calibration data gathering** (Cost: £213,930; 303 days)

**WPL: NK.** Panakeia will submit ethics approvals and request access to data (AppendixQu11). Subcontractors handle sample identification, IHC/ISH tests, scanning, annotation, and metadata collection, contributing site-specific calibration data. Pathology leads and clinical experts from Panakeia and subcontractors will finalise the study design for prospective validation.

**WP3: Site-specific calibration of PPB** (Cost: £241,053; 438 days)

**WPL: Dr. Salim Arslan** (SA). Conduct manual sanity checks, analyse image subsets, calibrate models, and share performance verification results. Calibrate PPB, troubleshoot system issues, monitor calibration, compile improvements, debug issues, and ensure data and model backups.

**WP4: Integration into sandbox environment** (Cost: £198,836; 360 days)

**WPL: Dr. Andre Geraldes (AG)**. Sandboxes set-up and infrastructure integration for Panakeia on partner infrastructure.

**WP5: Deployment. Validation and documentation** (Cost: £226,644; 162 days)

**WPL: AG.** Evaluate the product on a small blinded dataset from partners/subcontractors(2-3 sites), addressing security and system risks. Identify and fix issues, then deploy on prospective cases. Monitor ongoing product performance during this period.

**WP6: Clinical Performance Evaluation** (Cost: £29,432; 51 days)

**WPL: SA**. Statistical analysis on un-blinded prospective dataset by pathologists (site-specific) with subsequent confounding factor analyses (by Panakeia). Documentation of results for medical safety compliance in WP7.

**WP7: Budget Impact and Health Economic Analyses** (Cost: £99,060; 99 days)

**WPL: NK**. With PP and Operations manager, Leeds University (subcontractor) will create a budget impact model for the business case in NHS trusts and a Health Economics model for NICE HTA.

**WP8: Dissemination, communication and exploitation** (Cost: £125,821; 225 days)

**WPL: PP**. Documenting compliance for UKCA, CE-IVDD, NICE, and future CE-IVDR. Disseminate findings through conferences, drafts for peer-reviewed

journals, and general media. Refine post-project strategy for PPB commercialisation and HTA assessment.

**Management Approach**: Fortnightly WP reviews, resource and activity management by WP leader, with risk monitoring reported to PM via risk register(AppendixQu12). Adequate buffers in all WP's for potential overruns. Deliverables and milestones are key success measures. Applying PMBOK principles with rapid iterative agile development and test cycles.

Appendix Qu11.docx.pdf (opens in a new window) (/application/10114168/form/question/39396/forminput/108615/file/672922/download).

## Assessor feedback

### Assessor 1

All packages detailed are required to ensure that this project is rigorous and meets the objectives of the study. There is an appropriate project plan and Gantt chart

### Assessor 2

The project plan is overall aligned with the aims and objectives. The WPs appears to be relevant and are self-explanatory but additional specific information regarding the planned activities/tasks should have been included (particularly regarding the validation WPs with some information on the metrics for success to be used). Links and dependencies should have also been indicated. The timescale is challenging but the work is well spread across the 18 months. Costs and roles have been linked to the WPs

### Assessor 3

A balanced and well thought through project plan and execution strategy. Good detail - costings for project management are high but other considerations are reasonable.

### Assessor 4

The project work packages are outlined with the research category, lead partner and total costs provided for each one. The approach to project management is stated. The plan seems appropriate to the project objectives.

**Assessor 5**
The approach to project management is clearly stated and 8 workpackages are clearly described with costs and responsible leads. A Gantt chart is provided with Milestones although there are no clear interdependencies demonstrated between workpackages

## 12. Risks

Average score 8.4 / 10

**What are the main risks for this project?**

Panakeia identified and assessed project risks, likelihood and impact levels (**Risk-register, AppendixQu12**). Key risks are ranked and mitigation strategies developed. The WP1 lead & participants will advise the team on necessary changes. Risk register is updated in fortnightly review meetings.

**TECHNICAL:**

WP3,4,5:**Infrastructure limitations for calibration/validation/storage** (Medium). MITIGATION: Selected subcontractors have pre-existing digital pathology infrastructure meeting PPB requirements. Panakeia's scalable server resources facilitate large-scale validation, accommodating high image volumes. Existing infrastructure enables rapid scaling, with the team collaborating with AWS and GCP for technical support.

WP2,5:**Data provision delay** (High). MITIGATION: Pre-agreed requirements, ethical approvals, data transfer agreements in place where possible. Back-up NHS sites in pipeline. Weekly updates ensure timely data provision.

WP5:**Calibration falls short of expected results** (Low). MITIGATION: Selecting appropriate representative datasets for each site ensures robust calibration for maintaining performance and preventing sampling bias.

WP5,6:**Validation falls short of expected results** (Low). MITIGATION: Retrospective validation demonstrates high performance, mitigating risks. Site-specific calibration, performance verification, and power calculations for statistically-relevant cases in prospective validation further minimise risks. The risk register is managed by NK, with regular discussions with PP to mitigate research risks.

WP4:**Integration process is not proceeding as planned due to unexpected failures** (Medium). MITIGATION:Pre-assessment of the subcontractor infrastructure to ensure compatibility & integrability of PPB.

**COMMERCIAL:**

WP7:**Lack of budget impact case** (Low). MITIGATION: Preliminary evidence for budget impact models, using publicly available data and retrospective studies, demonstrate robust budget impact.

WP7:**Lack of health economic case** (Low). MITIGATION: Preliminary evidence for Health Economic models, using publicly available data and retrospective studies, demonstrate robust health economic case.

WP8:**New product enters the market** (Medium). MITIGATION: Panakeia's unique technology is backed by strong intellectual property protection and continued freedom-to-operate searches.

**MANAGERIAL:**

WP1:**Budget and time goals not met** (Medium). MITIGATION: Experienced team, detailed work-plan, regular meetings, tight budget control, and time-buffer in challenging WPs (WP2-6) to accommodate delays.

**FINANCIAL:**

WP1:**No match funding secured** (Low). MITIGATION: Panakeia secured commitments from investors to match eligible project costs. Additionally, pre-existing funds from previous VC raise and expected revenues from other offerings can be used for potential reinvestment if needed.

**CRITICAL INPUTS:** 1) Key personnel. 2) Knowledge of ML-models for pathology automation. 3) Histopathological data.

**REGULATORY CONSIDERATIONS:** In light of changing regulations (CE-IVDR), Panakeia will leverage its existing QMS to monitor and prepare relevant documentation necessary for maintaining UKCA & CE-IVD compliance. Company has in-house regulatory capabilities and established relationships with MHRA & BSI.

Appendix Qu12.docx.pdf (opens in a new window) (/application/10114168/form/question/39397/forminput/108621/file/672945/download).

### Assessor feedback

**Assessor 1**

There is a good risk register and appropriate mitigations. The main risks are associated with the sensitivity and specificity of the tests. While the team's

service appears to be able to integrate with various biological technologies, they should also ensure that their product fits into the workflow of the user.

**Assessor 2**
Good risk analysis featuring all the major risks (although regulatory approval has not been included). The risks are well evaluated and the mitigation strategies are detailed enough to provide confidence in their effectiveness.

**Assessor 3**
Good risk analysis with mitigation strategy. This section would have benefitted from scoring risks by categories: technical, operational, commercial, regulatory and or environmental (at least). Other factors such as clinical etc. would also been helpful.

**Assessor 4**
The key risks and uncertainties of the project are considered with appropriate mitigations. Relevant constraints or conditions on the project outputs are identified. The risk analysis seems realistic.

**Assessor 5**
Risks have been broken down clearly into managerial technical financial and commercial risks. Regulatory risks have been highlighted separately highlighting the importance of this aspect to the project. All key risks have been discussed with appropriate mitigations described. The risk register could have been improved by adding the expected risk score following mitigation to demonstrate the expected scale of the reduced risk

## 13. Added value

Average score 7.8 / 10

**How will this public funding help you to accelerate or enhance your approach to developing your project towards commercialisation? What impact would this award have on the organisations involved?**

**Public Funding Advantages:**

The IUK Investor-SME call **accelerates PPB's clinical adoption**, bridging the final real-world evidence gap needed for swift post-project commercialisation. **This funding will match VC investment**, boosting PPB's appeal to the NHS. Successful project outcomes will **unlock additional VC funding (Series A) and Pharma partnerships**, positioning Panakeia and the UK as global precision medicine leaders. The **projected 1,257% ROI** and 150 jobs created in the next 5 years highlight the UK's economic growth potential.

**Project Impact:**

Successful project delivery will be **transformative for Panakeia** and the NHS trusts (subcontractors), via accelerated access to groundbreaking technology. It will **open avenues for closing big-pharma partnerships** (in Panakeia's pipeline), paving the way for global product commercialization. The impact resonates not only in economic terms but also in solidifying the NHS' leadership within the global precision medicine landscape.

**Alternative Investment Considerations:**

**Panakeia's financing strategy blends dilutive and non-dilutive sources**, for generating real-world evidence needed for NHS adoption of. **Raising VC funds currently has limitations** (seed round, market conditions etc.), prompting the search for non-dilutive match-funding. The current grant is very timely with the project strongly aligning to call (Health and wellbeing). **Alternative plan involves seeking traditional funding & waiting for the right grant**, risking product development & market entry delays given lower investor appetite. This **poses a disadvantage**, allowing competitors to capture UK markets, hindering Panakeia's growth as a leading UK-based cancer diagnostic company.

**Existing and Potential Investment/Support:**

Panakeia has raised \>£4M in VC-funding till date. Panakeia is currently raising VC-funding to support ongoing operations and early commercialisation, including **match-funding for this project (commitments secured)**. Grant financing is imperative to sustain high-risk evidence generation critical for product commercialisation and achieving key series A milestones.

**Project Without Public Funding:**

**Without public funding, Panakeia faces significant setbacks**, leading to a 2-year delay (waiting for correct grants to support the project, NHS partners looking for alternative budgets) and losing the hospitals & pharma opportunities in the pipeline. Competitors, particularly two US companies, may exploit Panakeia's unique case, seizing UK markets, hindering its growth as a leading UK cancer diagnostic company.

**R&D Impact:**

**This project catalyses research expansion for all organisations involved**. Panakeia, as an SME, can continue developing novel ML approaches. Partners and subcontractors gain ROI on data/digital investments, accelerating research outputs & patient outcomes with AI. Biological questions spark new avenues, contributing to novel AI applications. Collaborative R&D fosters innovation and advances collective knowledge.

### Assessor feedback

**Assessor 1**

There are good arguments for grant funding, particularly to enter into the NHS marketplace. While there are indicators that this company will be able to sell into the NHS, it is still challenging and investors are often reluctant to have the NHS as the initial customer. Grant funding would incentivise innovation within the NHS as explained by the applicants.

**Assessor 2**

Although it is clear that support from InnovateUK will be beneficial for the project, the applicants have not specifically indicated the impact of InnovateUK support for the delivery/realization of the impact. Increase R&D opportunity (above and beyond what the applicants are already doing), leverage additional findings, accelerating delivery/approval has not been specifically mentioned and addressed. Impact in case of no funding has been mentioned but a 2-year delay appear relatively modest.

**Assessor 3**

A excellent impact statement for public funding.

**Assessor 4**

The arguments for public funding are good and justified. Alternative sources of support are described, with an indication of why they are discounted.

**Assessor 5**

Matched funding is committed from existing VC funds,. The applicants explain the benefit for non dilutive funding and the current issues in raisin additional VC funding. The successful completion of this project will enable greater VC funding for the commercialisation of the product The project will be delayed by

2 years without this funding although the applicants appear to be spending funds on other projects rather than concentrate on taking this lead application to market faster with existing funds. The funding will contribute to expansion of R&D spending

## 14. Costs and value for money

Average score 7.0 / 10

**How much will the project cost and how does it represent value for money for the team and the taxpayer?**

**18-month Experimental development project requesting 45% funding for lead SME**, with a total budget of £1.273 Million and **requested a grant of £572,720**.

Panakeia will fund the **remaining project costs (£700,000, 55%) through matched VC investment (commitments already secured)** required as a part of this call.

**Value For Money:**

Opportunity for innovative/revolutionary AI-product disrupting the BC market with multi-cancer applications.A globally-relevant solution with projected revenues exceeding £11M in 5 years, creating 150+ jobs, establishing initial dominance in BC market per the company's 5-year plan.

**Taxpayer Value:**

ML-based diagnostic solution enhances UK business competitiveness in digital healthcare, providing faster diagnostics, improved treatment stratification, accuracy, and cost-effectiveness for NHS pathology budgets (Qu7). Increased ROI for histopathology digitisation by early diagnosis & treatment stratification will lead to further ML applications within healthcare and diagnostics.

**Alternative Spend:**

**Given the strategic importance of this project for Panakeia**, this grant provides a unique, ***timely*** opportunity to work with leading key opinion leaders to realise a strategic partnership with a UK big-pharma company for global commercialisation & cementing UK's position in precision medicine/AI space. **Without the grant**, Panakeia would progress more slowly, face scale limitations, and rely on costlier funding sources, like venture capital and debt equity, **jeopardising the global opportunity**.

**Labour (£808,627):**

13 personnel (11 existing) over 348 days with involvement in project management, technical deployment, data provision, communication, testing/validation, and timely completion.

**Overheads (£161,725):**

20% of Labour costs

**Materials (£50,310):**

Infrastructure for PPB deployment and clinical validation.

**Work Package Cost Breakdowns:**

WP1: 288 days, Labour=£114,707;

WP2: 303 days, Labour=£130,428; Materials=£8,807; Subcontracting=£47,610; Travel & Subsistence: £1,000

WP3: 438 days, Labour=£193,538, Materials=£8,807;

WP4: 360 days, Labour=£154,945; Subcontracting=£11,902; Travel & Subsistence: £1,000

WP5: 162 days, Labour=£70,676; Materials=£32,711; Subcontracting:£107,123; Travel & Subsistence: £2,000

WP6: 51 days, Labour=£24,526;

WP7: 99 days, Labour=£32,956; Subcontracting=£59,512;

WP8: 225 days, Labour=£86,599; Subcontracting=£11,903; Travel & Subsistence: £10,000

**Post-project activities:**

Funded by VC investment and growing revenues. Successful commercial adoption will position the company for a £15Mn series A. Wider support: Clinical inputs, commercialization, reimbursement support leveraging existing networks.

**Subcontractors:**

NHS trusts costs (Labour and materials) associated with deployment and prospective evaluation of PPB. **Costs are based on historic costing and constitute 2-3 sites**.

**Assessor feedback**

**Assessor 1**

Even with a highly specialised team working on a complex project, the labour costs in this application are very high for a new company. Equally, the outcomes of this project could have excellent impacts on a wide range of stakeholders. On balance, the costs are acceptable.

**Assessor 2**

The overall resources requested appear to be on the expensive side also considering the short timescale and the prior work available. Although WP costs has been included, a better justification of the costs (in particular the labour costs with justification on the of 13 personnel and 348 days) should have been included (in this respect, detailed information of the planned tasks in the workplan would have provided some support). Remaining costs are provided by VC investment and it is not clear is any support will be provided by Panakeia

**Assessor 3**

A good justification statement, the only anomaly appears to be for WP1, project management that is c. 100K higher than similar projects.

**Assessor 4**

The project costs seem to be acceptable, but the justifications are not clear. The partners have indicated how they will finance their contribution. The balance of costs and grants between partners is not entirely satisfactory. Little information is offered about the value for money this project offers.

**Assessor 5**

A detailed breakdown of costs is provided, the majority of costs are on labour within Panakeia. The subcontractor costs are remarkably good value considering they are the essential aspect of the project delivery in terms of real world clinical data The need for both a project leader and project manager is not clear and would normally be combined in the same single role for a project like this. Likewise the need for a chief of staff in delivery of this specific project is not clearly justified.

# Terms and conditions

## Investor Partnerships terms and conditions

| Partner | Funding rules | Terms and conditions |
|---|---|---|
| PANAKEIA TECHNOLOGIES LIMITED (Lead) | Subsidy control | Innovate UK - Subsidy control (/application/10114168/form/terms-and-conditions/organisation/30145/question/39385) |

# Assessor feedback

**Assessor 1**
This is a highly innovative application that showcases the potential for AI-based companies to substantially improve the current healthcare dynamic, while maintaining quality assurances.

There is a specialised team who improve the chances of this highly technical project of succeeding in the marketplace.

**Assessor 2**
Strong proposal with an innovative approach aimed at addressing a well identified healthcare need. The team is well placed to deliver the proposed activities and the expected impact (on the business and the society) would be significant. Additional information regarding the planned activities and its costs in terms of labour days, would have been beneficial.

**Assessor 3**
A good project outlining important achievements leading to the current project for Breast Cancer for determining ER, PR & HER2 biomarker status. Pursuit of clinical evaluation and health economics are an important next step. Otherwise a strong team and excellent project to deliver next gen diagnostics for a high impact cancer.

**Assessor 4**
Good application with strong team. Need stronger commercial input. Also unclear on the costs and value for money and need more detailed plans on this.

**Assessor 5**
The applicants have produced a well written and detailed project proposal for their Breast cancer screening programme. The market analysis and route to market was very well described. A bit more detail on the project roles for individuals to justify their involvement would have been useful, especially with the labour costs being the largest part of the project costs within Panakeia.

The risk register could provide an expected risk score following mitigation to demonstrate the benefit of the mitigation.
